# Epidemiology Update of Hepatitis E Virus (HEV) in Uruguay: Subtyping, Environmental Surveillance and Zoonotic Transmission

**DOI:** 10.3390/v15102006

**Published:** 2023-09-27

**Authors:** Florencia Cancela, Romina Icasuriaga, Santiago Cuevas, Valentina Hergatacorzian, Mauricio Olivera, Yanina Panzera, Ruben Pérez, Julieta López, Liliana Borzacconi, Elizabeth González, Natalia Montaldo, Melissa Gaitán, Sandra López-Verges, Viviana Bortagaray, Matías Victoria, Rodney Colina, Juan Arbiza, Mabel Berois, Santiago Mirazo

**Affiliations:** 1 Departamento de Bacteriología y Virología, Instituto de Higiene, Facultad de Medicina, Universidad de la República, Montevideo 11600, Uruguay; fcancela@higiene.edu.uy (F.C.);; 2 Sección Virología, Instituto de Química Biológica, Facultad de Ciencias, Universidad de la República, Montevideo 11600, Uruguaynmontaldo@fcien.edu.uy (N.M.);; 3 Sección Genética Evolutiva, Instituto de Biología, Facultad de Ciencias, Universidad de la República, Montevideo 11600, Uruguayrperez@fcien.edu.uy (R.P.); 4 Departamento de Ingeniería Ambiental, Facultad de Ingeniería, Universidad de la República, Montevideo 11600, Uruguay; 5 Instituto de Ingeniería Química, Facultad de Ingeniería, Universidad de la República, Montevideo 11600, Uruguay; 6 Departamento de Virología y Biotecnología, Instituto Conmemorativo Gorgas de Estudios de la Salud, Panamá 0801, Panama; 7 Sistema Nacional de Investigación, Senacyt, Panamá 0801, Panama; 8 Laboratorio de Virología molecular, Departamento de Ciencias Biológicas, CENUR Litoral Norte, Sede Salto, Universidad de la República, Salto 50000, Uruguaymatvicmon@gmail.com (M.V.);

**Keywords:** Hepatitis E virus, subtype 3o, wastewater, Uruguay, molecular epidemiology

## Abstract

Hepatitis E Virus (HEV) infection is an emergent zoonotic disease of increasing concern in developed regions. HEV genotype 3 (HEV-3) is mainly transmitted through consumption of contaminated food in high-income countries and is classified into at least 13 subtypes (3a–3n), based on *p*-distance values from complete genomes. In Latin America, HEV epidemiology studies are very scant. Our group has previously detected HEV3 in clinical cases, swine, wild boars, captive white-collared peccaries, and spotted deer from Uruguay. Herein, we aimed to provide novel insights and an updated overview of the molecular epidemiology of zoonotic HEV in Uruguay, including data from wastewater-based surveillance studies. A thorough analysis of HEV whole genomes and partial ORF2 sequences from Uruguayan human and domestic pig strains showed that they formed a separate monophyletic cluster with high nucleotide identity and exhibited p-distance values over the established cut-off (0.093) compared with reference subtypes’ sequences. Furthermore, we found an overall prevalence of 10.87% (10/92) in wastewater, where two samples revealed a close relationship with humans, and animal reservoirs/hosts isolates from Uruguay. In conclusion, a single, new HEV-3 subtype currently circulates in different epidemiological settings in Uruguay, and we propose its designation as 3o along with its reference sequence.

## 1. Introduction

Hepatitis E virus (HEV) infection causes 20 million HEV infections and 70,000 deaths annually.

Particularly in industrialized non-endemic countries, it has been recently acknowledged as an important zoonotic agent of acute hepatitis [1].

The HEV virion consists of an icosahedral non-enveloped particle of 32–34 nm in feces and a “quasi-enveloped” particle of ~ 40 nm in circulating blood and culture supernatants [2]. The viral genome is a single-stranded positive-sense RNA molecule of approximately 7.2 Kb with a methyl guanosine cap at the 5′ end and a polyA tail at the 3′ end and contains three overlapping open reading frames (ORF1-3) [3]. ORF1 encodes a non-structural polyprotein, ORF2 encodes a capsid protein, and ORF3 is a multifunctional protein [4].

HEV belongs to the Hepeviridae family; the Orthohepevirinae subfamily and is divided into four genera: Avihepevirus, Chirohepevirus, Paslahepevirus, and Rocahepevirus. Paslahepevirus genus includes Paslahepevirus balayani and Paslahepevirus alci species, where the Paslahepevirus balayani species consists of HEV genotypes 1-8 (HEV1-HEV8) [5], which can be further divided into subtypes [6]. In developed countries, foodborne or direct contact with animal reservoirs, mostly domestic pigs and wild boars, are considered the main routes of HEV-3 and HEV-4 transmission. In this context, HEV causes generally self-limiting acute hepatitis, however, it can become chronic in immunocompromised and solid organ transplant patients, mainly related to HEV-3 infections [7].

HEV genotypes can be subdivided into subtypes and HEV-3 is considered the genotype with the highest genetic diversity, comprising 13 recognized subtypes and multiple lineages [6,8,9,10]. Recently, a distance-based criterion cut-off of 0.093 has been proposed to carry out consistent HEV-3 subtyping classification, simplifying the molecular epidemiology analysis [11].

HEV is an enteric virus that can be excreted as a non-enveloped particle in the feces of humans (wastewater) and animals (runoff from slaughterhouses, pig farms, or free-ranging animals). Waterborne HEV transmission can be direct, through contact with contaminated water, or indirect, by consumption of shellfish or crops irrigated with contaminated water [12].

In countries with poor sanitation conditions and inadequate treatment of sewage, waterborne fecal–oral route transmission of HEV-1 and HEV-2 can lead to epidemic outbreaks [12]. However, the transmission risk of zoonotic HEV-3 through water in developed or non-endemic countries is still under debate [12,13]. The HEV prevalence in water matrices worldwide is estimated to be about 15.14% in untreated wastewater, 3.81% in treated wastewater, and 7.46% in surface waters [12]. It has been suggested that HEV does not present high resistance to physical or chemical inactivators (UV, heat, chlorine, etc.) in contrast to other enteric pathogens such as the hepatitis A virus. Nevertheless, no other factors such as genotypes and quasi- or non-enveloped forms were considered in these studies [13].

Hepatitis E outbreaks or sporadic cases are reportedly annually in countries with limited access to clean water, in refugee camps, and in regions with humanitarian emergencies, and the World Health Organization (WHO) has released a technical report with several recommendations to plan and execute responses to contain these HEV waterborne outbreaks [14]. The main control strategies for these cases are divided into four categories of action: (1) prevention of exposure; (2) prevention of infection; (3) prevention of disease; and (4) prevention of death. Particularly, this manual strongly advises implementing diagnostics tests for HEV by trained personnel, since this disease is frequently mistaken for other forms of acute viral hepatitis because of specific tests being unavailable, which can result in an inadequate diagnosis and management of the patients [14].

In Uruguay, HEV-3 infections have been previously identified among human [15] and animal hosts (swine, wild boars, and white-collared peccaries) [16,17]. Extensive molecular and serological data have been obtained and recently we described a HEV-3 complete genome obtained from a solid organ transplant patient with chronic hepatitis E [18]. However, several knowledge gaps in the molecular epidemiology and modes of transmission of HEV remain. Though in the country HEV infection is notifiable within one week of the diagnosis, in the last decade as few as 25 cases have been officially recorded [19]. However, recent findings among blood donors evidenced a seroprevalence of 10% which suggests that the virus is being intensively and cryptically transmitted [20]. That might be explained, among other reasons, by the fact that hepatitis E is usually asymptomatic or subclinical, and is frequently indistinguishable from liver disease of a different viral etiology [21].

It is generally assumed that zoonotic events are the main source of HEV infection in developed regions and compiling molecular and epidemiological evidence supports this notion [22]. However, in less developed non-endemic areas this is more poorly understood and normally a source for human infection cannot be identified. 

Wastewater surveillance is a useful epidemiological tool that can provide data on the circulation of pathogens and has been used to evaluate infection trends in the community, monitor public health interventions, and make timely, evidence-based decisions to mitigate the impact of epidemic waves or outbreaks [23]. In Latin America, only a few HEV prevalence reports involving environmental and wastewater samples have been published [24,25,26,27]. Given the increasing trends of HEV seroprevalence in the region [28], developing efficient and standardized methodologies for the detection and characterization of HEV in the environment has been a real challenge. However, since the SARS-CoV-2 pandemic, WWS has undergone considerable improvement mainly due to an optimization of the concentrating and detecting protocols [29,30,31]. We have recently reported results from a country-wide-scaled WWS program for the early identification of SARS-CoV-2 VOCs [32].

The aim of this work was to reexamine the molecular epidemiology of zoonotic HEV in Uruguay by providing whole-genome data, updated sequence information with novel insights into the occurrence and the genetic features of HEV circulating in water matrices (wastewater and surface water), and more robust and detailed HEV-3 phylogenetic analyses. 

## 2. Materials and Methods

### 2.1. RNA Extraction and Reverse-Transcription Nested PCR (RT-nPCR) from Domestic Pigs Samples

Eighteen stool samples (10–15 gr) were collected in 2020 in sterile bags from a small-sized farm situated in Salto City, where the highest seroprevalence of HEV had been reported in a previous study [16]. Samples were kept on dry ice and sent to the laboratory where they were processed.

Pig fecal samples were resuspended at 10% *w/v* in sterile phosphate buffer saline (PBS) and vigorously vortexed. After centrifugation for 30 min, 8000× *g* at 4 °C, the supernatant was filtered to 0.22 μM. Total RNA was extracted with a Quick-RNA™ Miniprep Kit and treated with DNase I (Zymo Research Corp., Tustin, CA, USA) according to the manufacturer’s instructions. 

For the detection of HEV, RNA was subjected to RT-nPCR targeting a 330-bp region within ORF2 as previously reported [17]. PCR products were gel-visualized under UV light and amplicons of the expected size were sequenced in both directions by Macrogen Inc. (Seoul, Republic of Korea). 

The HEV-positive samples were filtered (0.45 μM) and viral particles were concentrated in a 30% sucrose gradient and ultra-centrifuged for 2:30 h at 100,000× *g*, 4 °C in a Sorvall™ WX+ Ultracentrifuge (Thermo Scientific, Waltham, MA, USA). The pellet was resuspended in 200 µL of PBS 1× and RNA was then extracted as mentioned above.

### 2.2. Wastewater and Surface Water Concentration, RNA Extraction and HEV Detection

**Figure 1:**
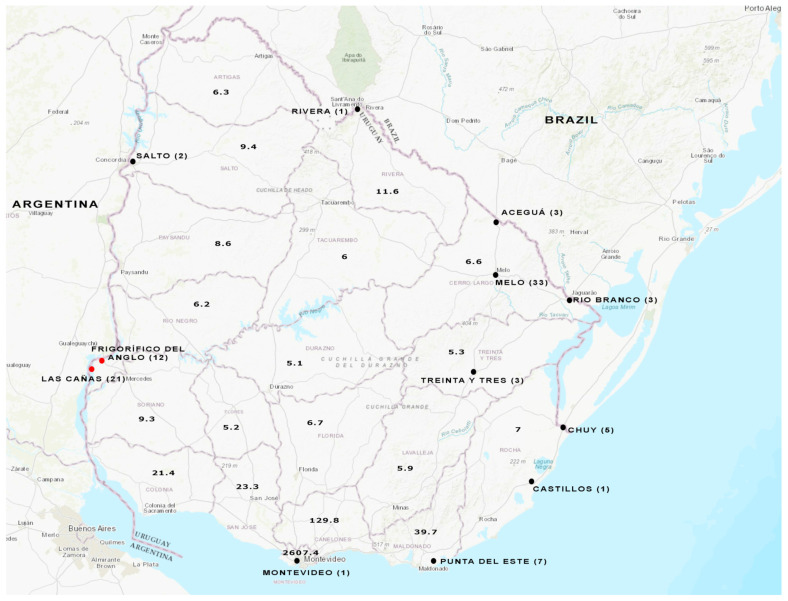
Water sampling from different cities throughout Uruguay. Wastewater samples were analyzed from the cities indicated with a black full circle. Surface water samples were analyzed from the places indicated with a red full circle. The number of samples studied is shown in brackets for each location. Population density (People/Sq Km) per department is indicated in the map, based on the values extracted from Anuario Estadistico Nacional 2019, 96^a^ version, Instituto Nacional de Estadística (INE), www.ine.gub.uy (accessed on 12 June 2023). The map of Uruguay was obtained from the USGS National Map Viewer (http://viewer.nationalmap.gov/viewer/, accessed on 12 June 2023).

Three sampling strategies were employed (Figure 1). Firstly, 4 h composite untreated samples were obtained from a timeline sampling carried out once a month from December 2020 to July 2021 (33 samples in total) at a wastewater treatment plant in Melo City. Secondly, a total of 5 samples (24 h composite) from four other cities (July 2021) across the country were included: Montevideo, Salto, Rivera, and Castillos. All these samples were part of the WWS program performed for SARS-CoV-2 [32].

Thirdly, 21 additional wastewater samples (4 h composite, 24 h composite, and/or simple) collected weekly during the period May–July 2023 were included. Sampling sites corresponded to the treatment plants of five other cities: Treinta y Tres, Rio Branco, Aceguá, Chuy, and Punta del Este.

Additionally, a total of 33 surface water samples from Las Cañas Beach and Frigorífico del Anglo area were included in the analysis [33]. As previously reported, these samples were collected monthly from May 2018 to April 2019. 

Wastewater concentration was performed as described [32]. Total RNA was extracted from the wastewater concentrates with the Quick-RNA™ Miniprep Kit (Zymo Research Corp., Tustin, CA, USA) according to the manufacturer’s instructions.

The RNA from surface water concentrates of Las Cañas and FA was already available [33].

HEV detection was performed with a broadly reactive one-step real-time RT-PCR [34] employing a specific Taqman^®^ probe with the SensiFAST™ Probe Lo-ROX One-Step kit (Bioline, London, UK).

A region within the ORF2 was amplified from the HEV-positive samples using a RT-nested PCR as previously described [15]. Amplicons of the expected size (958 bp) were sequenced by the Macrogen Inc. automatic sequencing service (Seoul, Korea) to perform phylogenetic analyses and construct nucleotide p-distance matrices.

### 2.3. Full-Length Genome Sequencing by Next Generation Sequencing (NGS), Sequence Analysis and Phylogenetic Reconstruction

RNA obtained from concentrated viral particles present in swine stool samples was subjected to NGS. For this, double-stranded cDNA (dscDNA) was generated with random primers using Maxima H Minus Double-Stranded cDNA Synthesis Kit (ThermoFisher Scientific, Waltham, MA, USA). dscDNA was amplified via Multiple Displacement Amplification (MDA) technology using a REPLI-g Mini Kit (Qiagen, Hilden, Germany) followed by purification and quantification using AMPure XP (Beckman Coulter, Brea, CA, USA) and a Qubit fluorometer (Qubit™ DNA-HS Assay kit, Thermo Scientific, Waltham, MA, USA), respectively.

Libraries were constructed from 50 ng of dscDNA using the Nextera DNA Flex Library Preparation kit (Illumina, San Diego, CA, USA) with dual indexing. Control quality libraries were performed on a Fragment Analyzer 5200 system (Agilent Technologies, Santa Clara, CA, USA) using the Standard Sensitivity NGS Analysis Kit (Agilent Technologies, Santa Clara, CA, USA). The library was sequenced on an Illumina MiniSeq Genomic Platform at Facultad de Ciencias (Universidad de la República, Montevideo, Uruguay) using a Mid Output Reagent Cartridge (300-cycles, 150 base-pair paired-end reads) and following standard Illumina protocols. 

Raw reads were demultiplexed automatically on the MiniSeq platform. Adapter/quality trimming and filtering were performed with the BBDuk plugin and clean reads were mapped to a previous Uruguayan hepatitis E genome (MW596896) using Geneious mapper (medium-low sensitivity) available in the Geneious Prime 2020.2.1 software (https://www.geneious.com accessed on 3 February 2022).

Sequences were assembled and annotated with SeqMan NGen^®^ Version 12.0 (DNASTAR. Madison, WI, USA).

Nested-PCR and subsequent sequencing of partial ORF1 and ORF2 [15] were performed to corroborate the NGS data.

The phylogenetic tree was reconstructed based on HEV3 full-length genomes using the neighbor-joining method with Tamura Nei as the substitution method, using Molecular Evolutionary Genetics Analysis (MEGA) v7 software [35]. Reference sequences of subtypes 3a to 3n according to Smith et al. [6] were retrieved from GenBank and included in the analysis. The substitution model that best fitted the data was obtained with MEGA v7 [35]. The robustness of the tree was determined via bootstrap v10 analysis (1000 replicates).

To further characterize the sequences obtained from water sources (wastewater and/or surface water), an additional analysis of HEV-3 subtypes was performed with a 768 bp region within the ORF2. A subset of previously reported human HEV partial-genome sequences from Uruguay were included [15]. The phylogenetic tree for the partial ORF2 region was reconstructed as described for the whole genome dataset.

Nucleotide p-distance matrices between Uruguayan strains and reference sequences of each HEV-3 subtype were constructed with MEGA v7 software for both the partial ORF2 region and full-length genomes.

## 3. Results

### 3.1. HEV Detection in Domestic Pig Stool Samples via RT-nPCR

Investigation of HEV was performed in swine fecal samples from a farm with elevated seroprevalence [16]. Three out of the 18 stool samples from the pigs were positive for HEV-RNA. Further sequence analysis of the partial ORF2 region grouped them within the HEV-3 genotype. 

### 3.2. Whole-Genome Analysis of a Swine HEV Strain

**Figure 2:**
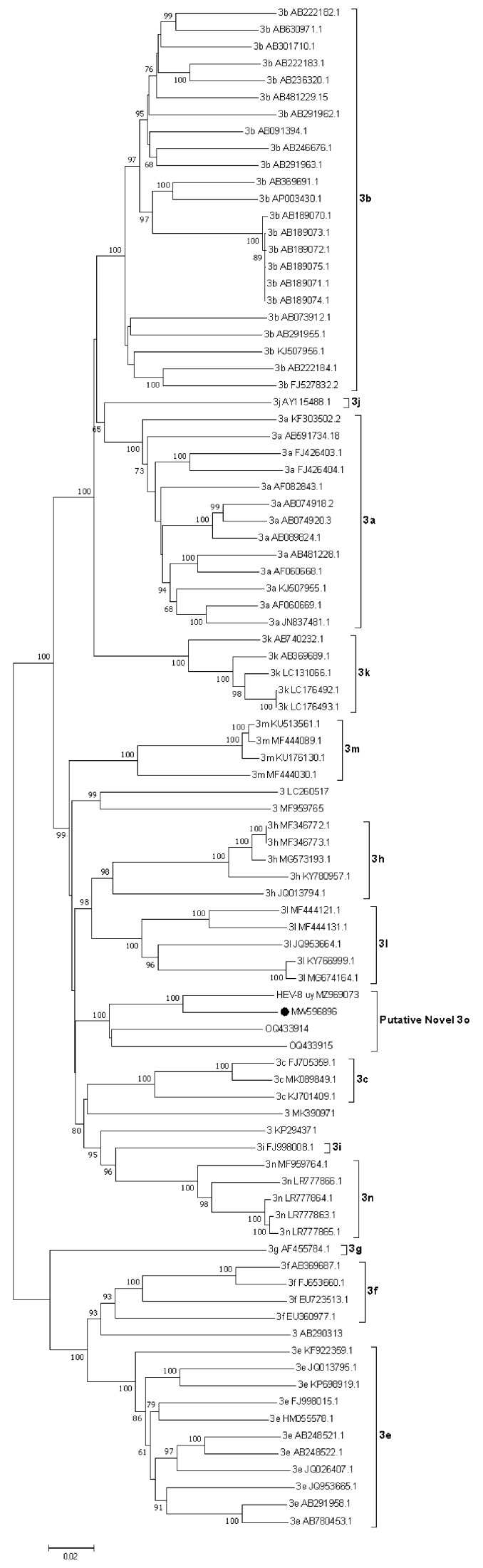
Phylogenetic reconstruction of HEV complete genomes for genotype 3. The tree was generated with the Neighbor-Joining algorithm using the Tamura-Nei parameters method employing Molecular Evolutionary Genetics Analysis (MEGA) v7 software. The robustness of the tree was determined via bootstrap analysis for 1000 replicates. Only values ≥60% are shown. Reference sequences for the subtypes 3a–3n and unclassified isolates are included. The putative new 3o subtype proposed for Uruguayan (MW598896 and MZ969073) and Brazilian sequences (OQ433914 and OQ433915) is shown and the proposed reference strain for this subtype (MW596896) is indicated with a black full circle.

NGS of the HEV-positive RNAs was successful in only one sample, HEV-8_uy. The whole genome sequence had 7082 nt and a 55.4% GC content. The analysis performed revealed a high percentage of identity (91.83%) with a human full-length HEV-3 strain recently described by our group (C1UY18, GenBank accession MW596896) [18]. Phylogenetic reconstruction grouped these strains together but according to bootstrapping analysis, they did not cluster in any of the known or assigned subtypes (Figure 2). For practical purposes, this cluster which also includes recently reported strains from Brazil [36] was named the putative 3o subtype in this study. Furthermore, the percentage of nucleotide identity between genomes from Uruguay (C1UY18, HEV-8_uy) and Brazil (PRsw1, RJ-sw1) within this cluster showed a range of 84.7% to 88.1%.

Given its extreme variability at the nucleotide level and the considerable amount of insertions and deletions observed among sequences, the hypervariable region was excluded from the analysis, as previously proposed [6]. 

The HEV-8_uy sequence was deposited in the GenBank database with the accession number MZ969073.

### 3.3. Analysis of Wastewater and Surface Water Samples

HEV RNA was detected via RT-qPCR in 10.87% of untreated wastewater samples (10/92), with Ct values ranging from 32.42 to 35.81 (Table S1). All the HEV-positive samples belonged from the 4 h composite sampling from Melo City. The partial ORF2 region was successfully amplified in two of these samples (named HE-67-WW and HE-112-WW).

**Figure 3:**
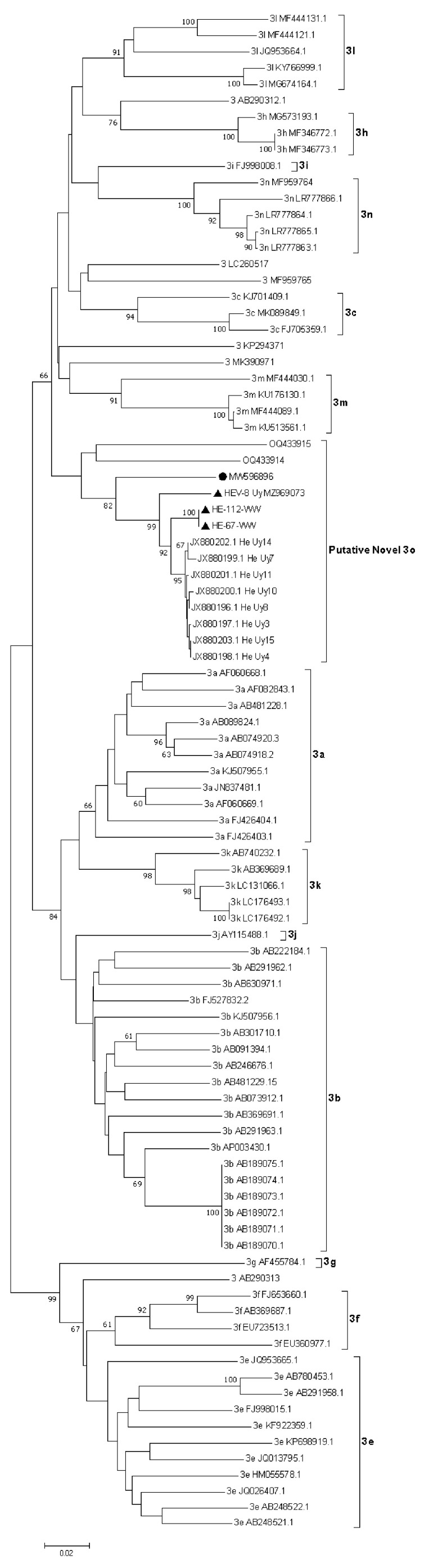
Phylogenetic tree based on a 719 bp fragment within ORF2. Tree reconstruction was performed using the Neighbor-Joining method with the Tamura-Nei model using Molecular Evolutionary Genetics Analysis (MEGA) v7 software. The robustness of the tree was determined via bootstrap analysis for 1000 replicates. Only values of ≥60% are shown. Reference sequences for the subtypes 3a–3n and unclassified strains are included. The HEV-3 wastewater sequences (HE-67-WW and HE-112-WW) and the swine complete genome HEV-8_uy (MZ969073) reported in this study are indicated with a black triangle. Proposed reference sequence for the novel 3o subtype is indicated with a black circle.

The phylogenetic analysis showed that the HE-67-WW and HE-112-WW strains were grouped in a separate cluster (putative 3o HEV-3 subtype) with previously reported partial and complete HEV-3 sequences detected in swine and human cases from Uruguay (Figure 3). 

HE-67-WW and HE-112-WW sequences were deposited in the GenBank database with the accession numbers OR267146 and OR267147.

On the other hand, HEV RNA was not detected in any of the surface water samples analyzed.

### 3.4. HEV Subtyping

To go further into the characterization of the HEV whole genomes and the wastewater sequences at the subtype level, p-distance matrices were constructed for the 768-nt ORF2 region and full-length genomes using reference strains (Table 1). We performed this analysis because it has been proposed and extensively used as the reference method for HEV-3 subtyping classification [6,11].

The analysis also included a set of reported human HEV ORF2 partial sequences from Uruguay for which no complete genome was available (Table 1) [15]. Comparison at the ORF2 region between the HEV-8_uy swine strain with human and wastewater isolates (all belonging to the putative 3o subtype) showed a percentage of nucleotide identity ranging from 94.7% to 95.4%. 

Using whole-genome analysis, Uruguayan samples were compared to the two recently reported HEV-3 swine sequences from Brazil (PRsw1-OQ433914 and RJsw1-OQ433915) [36] and subtype reference strains (3a–3n). Nucleotide sequence distance values of 0.118–0.125 and 0.132 to 0.177 were observed, respectively. 

Furthermore, a p-distance matrix constructed with the Uruguayan strains showed that the two complete genomes exhibited a p-distance value of 0.075. On the other hand, the p-distance values calculated for the partial ORF2 region from all 14 Uruguayan strains ranged from 0.000 to 0.096 (Table 2).

## 4. Discussion

HEV is a major cause of acute viral hepatitis worldwide and the only one that has an animal reservoir [37]. In developed areas, zoonotic seems to be the main route of infection of HEV-3 with infected pigs and/or wild boars as the main sources. The importance of the viral epidemiology of other routes and the role of potential animal hosts or reservoirs that could transmit the virus, including deer, rat, and livestock is still a matter of current debate [38]. There is a consensus that a better understanding of the transmission paths is needed especially for zoonotic HEV, in areas where a waterborne transmission is questioned. On the contrary, in less developed areas with heterogeneous socio-economic levels and non-optimal hygiene/sanitary conditions, the waterborne transmission of HEV-3 is likely to occur, as it is frequently detected in water matrices and aquatic environments [13]. 

Previously reported data by our group has shown that HEV infection occurs frequently in Uruguay, with a seroprevalence rate of 10% among blood donors, including three viremic individuals [20]. The fact that only a handful of cases have been reported in the last decade likely suggests a high rate of asymptomatic infection [39]. HEV infection has also been identified and characterized in domestic pigs, with an antibody and RNA prevalence of 46.8% and 16.6%, respectively, and in wild boars, indicating a widespread circulation of the virus in their natural reservoirs [16]. Additionally, we showed that HEV-3 could also infect and be transmitted among white-collared peccaries (New World pigs) and spotted deer (*Axis axis*) [17,40]. In this work, to provide novel insights into the molecular epidemiology and transmission patterns of HEV in Uruguay we reexamined the phylogenetic relationships with updated data and additional analyses.

First, the whole-genome comparison of the HEV strain identified in swine in this study (named HEV-8_uy) exhibited a high nucleotide sequence percentage of identity with a previously reported strain detected in 2019 in a chronically infected patient [18]. Even though the source of infection could not be identified in any of the human cases in our country, this finding confirms previous data [16] and raises concern about the zoonotic role that domestic pigs are playing in the dynamics of hepatitis E. In addition, these sequences were grouped in a monophyletic cluster within HEV-3 with two recently published Brazilian swine HEV-3 genomes from the Paraná and Rio de Janeiro states (OQ433914-OQ433915) [36]. Surprisingly, this cluster could not be assigned to any known (or currently unassigned) HEV subtype according to bootstrap analysis. To confirm this finding, p-distance matrices were constructed covering all assigned (3a–3n) subtypes’ reference strains and those sequences not assigned to any subtype of HEV-3. According to the proposed criteria, a cut-off value of 0.093 is usually applied to define subtypes [11] and the sequence distance values obtained here (0.132 to 0.180) suggest that the Uruguayan HEV-8_uy and MW596896 strains indeed comprise a novel HEV-3 subtype. Thus, we propose the sequence C1UY18 (GenBank accession number MW596896) to be the reference sequence for this new 3o subtype, as it was the first one reported for this cluster. On the other hand, though p-distance values (0.118–0.125) between OQ433914 and OQ433915 and the Uruguayan sequences exceed the cut-off value, and strictly they could belong to two different new subtypes, this is not always an absolute criterion, and considering that the four strains come from neighboring countries in South America, we suggest that they can be initially classified into the same subtype.

As a second goal, in this study, we investigated the presence of HEV in wastewater and surface water to provide additional data on the epidemiology of the infection and the viral transmission patterns in the country. Wastewater-based epidemiology and surveillance of aquatic environment matrices have been employed as cost-effective tools for monitoring the circulation of pathogens to make public health decisions [23] but they can also be useful for assessing transmission chains and investigating epidemiologic links during outbreaks or clusters of cases [24].

HEV RNA was detected in 10.87% (10/92) of the wastewater samples collected. In a regional context, this frequency of detection is markedly higher than the 1.6% reported in environmental samples from northeast Argentina (Salta City) [26] but lower than the detected in sewage samples from western Argentina (Mendoza City) (22.5%) [26] and in wastewater from Colombia (16.7%). Of note, all HEV-positive samples belonged to Melo City. The wastewater treatment in Melo City consists of a separate system in which domestic sewage and rainwater runoff do not mix, nevertheless, this is a leaky system where rainwater enters the sewage collector and the effluent is consequently diluted when precipitation occurs [41]. Considering that the wastewater treatment system in this city is similar to others (Rivera, Salto, and Castillos) and that there are no significant social or environmental differences between the places studied, it is striking that HEV was only detected in Melo City. However, as previously reported, SARS-CoV-2 had been detected in all these cities throughout the WWS program [32]. The analysis of the partial ORF2 sequence of the HE-67-WW and HE-112-WW strains identified in wastewater demonstrated that they share a high nucleotide identity between them and are clustered within the novel proposed 3o subtype of HEV-3. In addition, as expected, they exhibited a marked nucleotide divergence with other described HEV-3 subtypes. Altogether, all these findings support previous data that suggested that HEV had a recent introduction in Uruguay and likely from a single geographic origin [42], which is in sharp contrast to other South American countries [43,44] Significant research efforts have been made to obtain additional complete genomes from Uruguay through different enrichment methods, but the low HEV titers in the sample and the lack of an efficient cell culture system have severely hampered the results.

Even though analysis of partial sequences is in general not suitable for HEV-3 subtyping, it has been proposed and accepted that the ORF2 region could eventually be used for this purpose, since it largely reflects the data obtained with whole-genome analysis [45]. The 3d subtype was designated based exclusively on three partial 304 bp regions within ORF2 from swine samples [6,45,46]. Furthermore, the phylogenetic and nucleotide analysis performed in this work involving partial HEV ORF2 from Uruguayan strains, including several previously identified in human cases [15], showed similar p-distance values and phylogenetic tree topology to the analysis performed with full-length genomes. Unfortunately, ORF2 sequences from white-collared peccaries and spotted deer were not available for this study and this is a limitation in terms of exploring potential transmission paths involving other ecological reservoirs/hosts. However, previous analyses carried out with ORF1 partial sequences had shown close phylogenetic relationships with sequences of human origin [17,40].

In summary, we provide updated data on the molecular epidemiology of zoonotic HEV-3 in Uruguay and propose the designation of a new subtype 3o with its reference sequence. The data presented here suggest that our country is unique in terms of the molecular epidemiology and transmission paths of HEV, in that a single circulating subtype has been identified in humans, animal reservoirs and hosts, and environmental samples. Furthermore, despite the environmental and domestic pig samples being collected about 10 years after the first human cases were described [47], low genetic heterogeneity is still observed between strains within this new subtype, suggesting little diversification over time. On the other hand, waterborne transmission of HEV remains to be confirmed and further investigated by assessing virus particle stability and infectiousness using standardized cell culture systems [48].

## Figures and Tables

**Table 1:** Summary of *p*-distances between Uruguayan strains, proposed here as a new subtype 3o vs. reference subtypes (3a–3n), and unassigned strains comparing complete genomes and partial ORF2 region. Brazilian sequences (putative 3o subtype) are indicated (*).

Accession Number	Subtype	Complete Genome ^a^	Partial ORF2 ^a^
AF082843	3a	0.147–0.150	0.131–0.141
AP003430	3b	0.144–0.145	0.133–0.146
FJ705359	3c	0.136–0.140	0.134–0.147
AF296165-7	3d ^b^	-	0.132–0.149
AB248521	3e	0.171–0.177	0.137–0.163
AB369687	3f	0.174–0.176	0.143–0.158
AF455784	3g	0.169–0.174	0.160–0.170
JQ013794	3h	0.132–0.137	0.114–0.128
FJ998008	3i	0.132	0.120–0.136
AY115488	3j	0.151–0.155	0.133–0.143
AB369689	3k	0.146–0.148	0.130–0.136
JQ953664	3l	0.136–0.139	0.124–0.143
KU513561	3m	0.134–0.135	0.120–0.143
MF959764	3n	0.133–0.137	0.120–0.146
PRsw1 OQ433914 *	Putative 3o	0.118–0.119	0.110–0.126
RJ-sw1 OQ433915 *	Putative 3o	0.121–0.125	0.115–0.122
KP294371	Unassigned	0.136	0.126–0.136
LC260517	Unassigned	0.142–0.146	0.117–0.148
MF959765	Unassigned	0.134–0.141	0.143–0.153
MK390971	Unassigned	0.142–0.145	0.116–0.136
AB290313	Unassigned	0.175–0.180	0.150–0.168

^a^ minimum and maximum values are shown. ^b^ Only 304 bp fragment within ORF2 is available for this subtype.

**Table 2:** Summary of *p*-distances between Uruguayan strains comparing complete genomes and the partial ORF2 region.

Strains	Subtype	Complete Genome	Partial ORF2 *
Uruguayan isolates	Putative 3o	0.075	0.000–0.096

* Minimum and maximum values are shown.

## Data Availability

The sequence data presented in this study are openly available in Genbank (https://www.ncbi.nlm.nih.gov/genbank accessed on 18 September 2023).

## Supplementary Materials

The following supporting information can be downloaded at: https://www.mdpi.com/article/10.3390/v15102006/s1, Table S1: Real-time PCR Ct value, date, and sampling site from Uruguayan HEV-positive wastewater samples. The two samples that were amplified by RT-nested PCR are indicated.
