# Supplementary material for: Epidemiology Update of Hepatitis E Virus (HEV) in Uruguay: Subtyping, Environmental Surveillance and Zoonotic Transmission
Source: Viruses. 2023 Sep 27;15(10):2006. doi: 10.3390/v15102006 (PMC10612089; doi:10.3390/v15102006)
Supplement: Supplementary file 1 [file viruses-15-02006-s001.zip › viruses-2618304-supplementary.pdf]

Table S1. Real-time PCR Ct value, date, and sampling site from Uruguayan HEV-positive wastewater samples. The two samples that were amplified by RT-nested PCR are indicated (*).

Sample Name	Date	Ct Value	Sampling Site
HE-1-WW	November 2020	35.81	Melo
HE-58-WW	February 2021	32.42	Melo
HE-63-WW	March 2021	33.84	Melo
HE-67-WW *	March 2021	35.19	Melo
HE-75-WW	April 2021	32.90	Melo
HE-87-WW	April 2021	34.10	Melo
HE-88-WW	May 2021	34.70	Melo
HE-112-WW *	June 2021	35.71	Melo
HE-120-WW	June 2021	32.53	Melo
HE-125-WW	June 2021	34.61	Melo
